# Autism Spectrum Disorder: Neurodevelopmental Risk Factors, Biological Mechanism, and Precision Therapy

**DOI:** 10.3390/ijms24031819

**Published:** 2023-01-17

**Authors:** Ling Wang, Binquan Wang, Chunyan Wu, Jie Wang, Mingkuan Sun

**Affiliations:** The Key Laboratory of Modern Toxicology, Ministry of Education, School of Public Health, Nanjing Medical University, Nanjing 211166, China

**Keywords:** autism spectrum disorder (ASD), ASD animal model, neurobiological mechanisms, neurocircuit mechanisms, therapeutic strategies

## Abstract

Autism spectrum disorder (ASD) is a heterogeneous, behaviorally defined neurodevelopmental disorder. Over the past two decades, the prevalence of autism spectrum disorders has progressively increased, however, no clear diagnostic markers and specifically targeted medications for autism have emerged. As a result, neurobehavioral abnormalities, neurobiological alterations in ASD, and the development of novel ASD pharmacological therapy necessitate multidisciplinary collaboration. In this review, we discuss the development of multiple animal models of ASD to contribute to the disease mechanisms of ASD, as well as new studies from multiple disciplines to assess the behavioral pathology of ASD. In addition, we summarize and highlight the mechanistic advances regarding gene transcription, RNA and non-coding RNA translation, abnormal synaptic signaling pathways, epigenetic post-translational modifications, brain-gut axis, immune inflammation and neural loop abnormalities in autism to provide a theoretical basis for the next step of precision therapy. Furthermore, we review existing autism therapy tactics and limits and present challenges and opportunities for translating multidisciplinary knowledge of ASD into clinical practice.

## 1. Introduction

Autism is a collection of genetically variant neurodevelopmental disorders that manifest as early social intercourse dysfunction and impaired repetitive behaviors and interests [1]. Based on estimates from the Centers for Disease Control and Prevention’s Autism and Developmental Disabilities Monitoring Network, approximately 1 in 44 children are diagnosed with an autism spectrum disorder (ASD) [2]. The prevalence of autism is over four times greater among boys than girls, and it commonly has co-occurring conditions, including epilepsy, depression, anxiety, and attention deficit hyperactivity disorder, as well as challenging behaviors such as sleep and self-harm [3]. Autistic individuals have atypical cognitive deficits, like impaired social cognition and perception, executive dysfunction, and atypical perception and information processing. These features are underpinned by atypical neurodevelopment at the systems level [4].

Both genetics and environmental factors early in development play a vital role in the etiology of autism [5]. Genetic variation in genes dramatically increases ASD risk. Features of autism may be detected in early childhood, but the diagnosis of autism is usually not made until much later. Early diagnosis requires a joint multidisciplinary assessment, and targeted behavioral interventions and pharmacological treatment can only somewhat reduce the social impairment and emotional instability-induced aggression and decrease the complications, but cannot completely cure them. Current research has not identified clear neuropathological markers of autism that can provide a basis for diagnostic criteria, and at this stage, it is speculated that abnormal behavior in autism is associated with alterations in emerging properties of brain function. Thus, studying the physiological mechanisms and potential pathogenesis of brain circuits is crucial for future diagnostic treatments.

**Figure 1:**
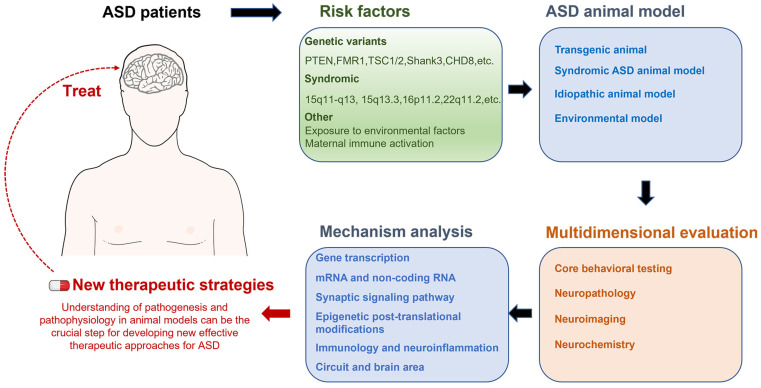
Translation cycle of ASD research. Epidemiological studies of ASD patients can identify abnormal genes and underlying risk factors, including genetic mutations, copy number variations (CNVs), environmental exposures, and maternal immune activation during pregnancy. Based on these data, different animal models will be developed and characterized for their variations. A comprehensive analysis of animal models combined with human pathology to understand the pathogenesis of ASD holds promise as a new therapeutic strategy to feed back to patients.

Here, we briefly overview advances made using transgenic mouse models as well as altered environmental factors to investigate ASD neuropathology. A comprehensive approach was used to analyze pathological correlations between ASD animal data and ASD populations. The molecular biological mechanisms and neural circuits associated with autism spectrum disorders were also integrated to explore further the critical circuit elements and underlying molecular mechanisms involved in each set of symptoms of ASD disorders. Finally, the progress and shortcomings of current ASD treatment options are summarized, as are the prospective difficulties of recent autism research and future development plans (Figure 1).

## 2. Technological Advances in Animal Models of Autism

Autism is a lifelong neurodevelopmental disorder with a vital hereditary element, but environmental factors, including toxicants, insecticide, infections, and medications, have been familiar to contribute to autism susceptibility. Current animal models of ASD include genetic animal models, CNVs -induced syndrome ASD animal models, idiopathic animal models and environmentally induced types, which have different advantages and individual limitations in generating ASD among different models [6,7]. We can improve drugs and optimize treatment regimens by thoroughly analyzing the mechanics of various animal models. Meanwhile newly developed devices facilitate combination of valproic acid (VPA) with other techniques to probe the neural basis of complex behaviors associated with ASD (Table 1).

### 2.1. Genetic Animal Models

Based on family and population studies, ASD heritability is about 50% that of mental illness and is even higher in identical twins [7]. Using present techniques, the genetic cause of the presence of ASD is clear in approximately only 20% cases [10]. In this part, we outline the neuropathological abnormalities reported in animal models following various gene ablation and assess the importance of transgenic models in ASD.

Neurexins (NRXNs): NRXN genes encode α and β-neurexin proteins, which assume a significant part in synaptic adhesion, differentiation and maturation as presynaptic binding partners of NLGN [7,8,9]. NRXN1, one of the susceptibility genes for ASD, has point mutations that are often associated with loss of function [66]. NRXN3 is also correlated with abnormal ASD function [67]. KO animals with all three NRXN genes knocked out or double knockout of NRXN1/2 were observed to have reduced inhibitory synapses in the brainstem and cortex. Contact protein-associated protein 2 (CNTNAP2), a member of the NRXN family, encodes contact protein-associated protein-like 2 (CASPR2). recessive mutations or chromosomal inversions in CNTNAP2 have been observed in ASD individuals. CASPR2 is required for dendritic arborization, stabilization of dendritic spines, and α-amino-3-hydroxyl-5-methyl-4isoxazole-propionic acid (AMPA) receptor trafficking. CNTNAP2 knockout mice also showed abnormalities in the corpus callosum and in somatosensory cortex neuronal migration.

Neuroligins (NLGNs): Trans-synaptic complexes formed by postsynaptic NLGNs and presynaptic neurexins are considered to facilitate synaptic stability. Different compositions of these cell adhesion molecules have been associated with formation of glutamate or γ-aminobutyric acid (GABA) synapses. MRI scans revealed that Nlgn3-knockout mice have a smaller brain volume than controls [12]. Nlgn3 knock-in (KI) mice showed increased postnatal turnover of excitatory spines in layer II and III pyramidal neurons of the prefrontal cortex and elevated expression of saccadic GABA transporters in somatosensory cortical neurons, but no changes in inhibitory synapse counts or ultrastructure [11,13]. The volume of multiple brain regions is reduced in Nlgn3^R451C^ KI mice that exhibit the Nlgn3^R451C^ mutation seen in persons with ASD [14]. The Nlgn3^R704C^ KI animal model, mimics an ASD-related mutation, demonstrates reduced AMPA receptor-mediated hippocampal neurotransmission but no similar reduction for N-Methyl-D-aspartic acid (NMDA) or GABA, perhaps due to increased AMPA receptor internalization [68].

SH3 and multiple ankyrin repeat domains protein 3 (SHANK3): SHANK3 is a postsynaptic density (PSD) protein that interacts with and binds with several ionotropic and metabotropic glutamate receptors. ASD and Phelan-McDermid syndrome are linked to SHANK3 mutations [15,18]. SHANK3 mutations and chromosomal rearrangements at 22q13.3 can cause Phelan-McDermid syndrome [69]. It has been suggested that SHANK3-deficient heterozygotes are associated with the defects observed in ASD and Phelan–McDermid syndrome [70]. Consistently, the current work shows that mouse models having distinct SHANK3 isoforms exhibit ASD behavior [71]. Models that ablate the full-length SHANK3 isoform by deleting exons 4–9 show decreased glutamate receptor 1 immunoreactive spots in hippocampal CA1 [19]. In SHANK3/- mice lacking Shank 3α and β dendritic length and complexity were increased, while PSD length and spiny neuron thickness were lessened. The striatum of KO mouse models disrupting all SHANK3 isoforms show abnormal spine density and PSD. Similarly, reinstating SHANK3 expression in mature SHANK3 deficient mice has been demonstrated to salvage dendritic spine loss and excitatory synaptic function in the striatum [69]. Finally, SHANK3 is critical in coordinating integration of the numerous glutamate receptors of the PSD and linking synaptic signaling to spinal movements.

Tuberous sclerosis complex 1/2 (TSC1/2): Tuberous sclerosis complex (TSC) is an autosomal dominant disorder with a feature of benign tumor nodules in a variety of organs and an elevated risk of malignancy [72]. The prevalence of ASD among TSC mutation carriers is between approximately 36% and 50% [47]. In TSC patients, abnormalities in neuronal migration and differentiation in and around cortical nodules have been observed [73]. Research in TSC2 mutant subjects suggest that cortical nodules have abnormal neurons and aberrant stratification, along with cell loss leading to hippocampal sclerosis and cerebellar atrophy [20,21]. Tsc1 knockout in forebrain pyramidal neurons has little effect on somatic cell size or dendritic shape but increases the spine density of temporal lobe cortical neurons. The spine density and pruning of Tsc2+/− mice layer V neurons in the temporal cortex increased with age. In Tsc2+/− Eker rats, hippocampal neurons show an increase in spine length but a decrease in spine width and number of excitatory synapses. Researches have shown that TSC1/2 gene mutations lead to abatement of its inhibitory effect on mammalian rapamycin (mTOR) protein, causing misregulation of mTOR signaling [22].

Fragile X Mental Retardation 1 gene (FMR1): Fragile X is generated by amplification or uncommon point mutation in the promoter of cytosine-guanine-guanine trinucleotide in the fragile X mental retardation gene [74]. Fragile X syndrome mental retardation protein (FMRP) is an RNA-binding protein that modulates synaptic plasticity by interacting with specific mRNAs in the brain [23,24]. Furthermore, roughly 22% of FMR1 mutation carriers and 30% of men in this category match the ASD diagnostic criteria [19]. It was found that subjects with fragile X syndrome had increased spine density and length in the temporal lobe and visual cortex, predominantly in the immature spine. In the visual cortex of adult FMR1−/− mice, there were more immature spines and less mature spines [25]. Nevertheless, the opposite is true in CA1. FMR1−/− mice have increased spine density in the somatosensory cortex 4–7 days after birth. However, it is not present in the hippocampus. The above experimental results consider that FMR1 has brain region-specific effects in synaptic maturation. It has also been observed that FMR1 shows varying effects on synaptic development with age. Hippocampal neurons in FMR1 KO pups are dominated by the occurrence of short spines, while adult mice have more occurrence of long spines. The variation in spine morphology observed may be caused by changes in spinal flip. Adult FMR1 mice exhibit a higher rate of spine renewal in the visual cortex. The neurological and behavioral damage in FMR1 knockout mice can be rescued to some extent by removing p70 s6 kinase 1 or by treatment with polyunsaturated fatty acids [65]. Pietropaolo’s study revealed that daily supplementation with omega-3 fatty acids (n-3 PUFA) also improved social interaction, emotional and non-spatial memory, and normalized some of the symptoms in FMR1-Ko mice. It caused a reduction in some neuroiflammatory changes in the brain.

Methyl-CpG-binding protein 2 (MECP2): MECP2 gene mutations lead to Rett syndrome, an X-linked neurodevelopmental disorder primarily affecting females [75]. More than 61% of Rett syndrome patients match the criteria for ASD, including repetitive hand movements, social withdrawal, and loss of verbal communication [76,77]. Many human symptoms are generalized by mouse models lacking in MECP2, making them a suitable experimental paradigm for exploring the underlying processes of ASD behavior. Patients with Rett syndrome have reduced brain size, smaller neurons, increased neuronal accumulation, and reduced neuronal dendritic complexity in frontal and motor cortex layers III and V. Some mouse models have shown neocortical thinning. An increase in cell density and smaller neuronal cell bodies were noticed in several brain regions [28,78]. In MECP2-deficient mice, the spinal heads in the dentate gyrus and hippocampal CA1 regions are smaller, and axonal direction in the motor cortex is altered. These changes appear to result from the delayed neuronal growth and synapse formation induced by MECP2 haploinsufficiency, a developmental defect that does not improve with age. MECP2 deficiency leads to fewer glutamatergic synapses and higher baseline levels of AMPA, indicating an activity-dependent failure of synaptic receptor transport [29]. Mutant mice encoding the transcriptional regulator MECP2 gene present autism-like behavioral traits typical of Rett syndrome. Guy et al. found that re-expression of the MECP2 gene manipulated by gene-editing techniques in a mouse model of autism reversed behavioral changes similar to autism, as well as the typical neurological abnormal symptoms of Rett syndrome [65].

Emerging potential genetic models of ASD: In the following discussion, several models are ASD-related genetic models emerging in recent years, and although some of following have been validated for the ASD phenotype, relevant neuropathological data are less available. Thus, there is growing interest in potential emerging transgenic animal models.

The chromodomain helicase DNA-binding protein 8 (CHD8) gene encodes a chromatin-modified gene on chromosome 14q11.2, which has been identified as a high-risk gene for ASD. In a cohort of approximately 6000 autistic patients, 0.2% had specific ab initio CHD8 mutations [30]. Patients with CHD8 mutations have head-size differences, as well as presenting developmental delays, cognitive impairment, motor deficiencies, and anxiety [79,80]. CHD8 has been found to mediate the transcription of ASD risk factors in human neural progenitor cells, as well as brain development pathways including neuronal differentiation, synaptic development, cell adhesion, and axonal guidance [30]. In P23-25, a 7-nucleotide deletion in exon 1 resulted in CHD8 single-fold resistant (CHD8+/−) animals with somewhat reduced social interaction and diminished preference for social novelty. Relevant MRI analysis indicated that CHD8+/− mice have larger brain volumes than wild-type controls [31]. CHD8 exon 5 was deleted in order to create a strain of CHD8+/del5 mice that showed transcriptional changes in neurodevelopmental disease pathways such as neurogenesis, synaptic processes, and neuroimmune signaling. The anterior cortical and neocortical areas were increased after birth [32].

Sodium channel power-gated type II subunit (SCN2A) mutations are correlated with epilepsy, intellectual disability, and ASD without epilepsy [81]. Generally, mutations altering neuronal sodium channel structure, function, or expression lead to epilepsy and neurological disease, and SCN2A stop codon mutations lead to termination of protein translation in autism [82]. It has been estimated that 7.5 out of every 100,000 births are diagnosed with a pathogenic mutation in SCN2A. In 50% of patients diagnosed with SCN2A syndrome, symptoms are similar to the more familiar forms of ASD, including reduced social interaction and repeated behaviors [83]. Adult subtypes of Scn2ain mice showed neuronal hyperexcitability [84]. Mice with the Scn2aGAL879-881QQQ mutation exhibited neuronal loss and glial hyperplasia in the hippocampus.

Synaptic GTPase-activating protein 1 (Syngap1) is a Ras-GTPase-activating protein that exists in the postsynaptic density of glutamatergic neurons and participates in dendritic spine formation, glutamate receptor transport, and synaptic function [85]. Syngap1 mutations are linked to various neurodevelopmental diseases, including non-syndromic intellectual disability and ASD [86]. Compared to WT mice, Syngap1 deficiency resulted in early dendritic branching, premature pruning, and larger dendritic spines in somatosensory cortical pyramidal neurons at key times in development. The density of mushroom spines in the hippocampus CA1 of Syngap1+/− mice was enhanced compared with WT mice, but there was no change in the density of fine or thick spines. Selective induction of Syngap1 deficiency in GABAergic neurons causes reduced presynaptic neuron density in somatic cells and decreased axon terminal branching of interneurons on cortical interneurons [34,35]. Syngap1 expression is not expressed in the development of glutamatergic neurons in the forebrain, whereas it is expressed in GABAergic neurons, causing cognitive impairment in mice [19].

Glutamate receptor ionotropic NMDA2B (Grin2b) is linked to epileptic encephalopathy, ASD, and other neurological disorders in which Grin2b haploinsufficiency is often an important factor [45,87]. GluN2B deletion disrupts protein-dependent homeostatic plasticity, according to in vitro electrophysiological investigations. In vitro inhibition of Grin2b causes delayed migration of cortical neurons, as well as increases in dendritic length and branching. The involvement of GluN2B in modulating synaptic maturation has been established in rat cortical and spinal cord co-cultures, with synaptic elimination reduced when postsynaptic GluN2B is lacking. Dendritic spine density is decreased in mice with conditional GluN2B ablation in CA3 pyramidal neurons and CA1 pyramidal neurons [88].

### 2.2. Syndromic ASD Animal Models Caused by CNVs

15q11-q13 deletions and 15q13.3 microdeletions: Genomic deletions within the chromosome 15q11-13 locus cause different neurodevelopmental syndromes. Prader-Willi and Angelman syndromes are the most common, with Prader-Willi syndrome caused partly by a deletion on the paternal copy of chromosome 15q11.2-q13, uniparental dimorphism, or imprinting center defects. Patients with Prader-Willi syndrome have abnormal dentate and olivary nuclei distribution, dentate nucleus neurodegeneration, cerebellar ectopia, expanded ventricles, volume reduction in the parieto-occipital lobe, numerous cerebellar gyri in the lateral fissure, and smaller cerebellum and brainstem. Angelman syndrome is caused by maternal copy deletion, chromosome 15q11.2-q13 abnormalities or the E6-AP ubiquitin protein ligase (UBE3A/E6AP) gene mutation [89]. Approximately 34% of those affected exhibit autistic-like characteristics. Angelman syndrome patients have smaller brains, cerebellar atrophy, and reduced white matter integrity. In maternal illness models, many and thick neural spines are found, as well as decreased presynaptic GABA vesicle density at inhibitory and excitatory synapses [47,48]. 15q13.3 deletion is associated with an increased risk of ASD, intellectual disability, schizophrenia, and epilepsy [50]. A newly produced heterozygous D/+ mouse model with a homozygous microdeletion had larger brains and lateral ventricles in adulthood, and this microdeletion has also been linked to head enlargement in humans.

16p11.2 deletion and duplication syndromes: Duplications and deletions of the 16p11.2 gene can result in ASD and other neurological problems [90,91]. According to recent research, 20% of patients with duplications and 16% with deletions had ASD-like behavior [51,52]. Subjects with 16p11.2 deletions displayed large head malformations, whereas duplicated carriers displayed small head deformities [92]. Compared to WT controls, the 16p11.2 deletion model in mice results in decreased brain weight, cortical dimensions and disrupted cortical compartmentalization [93]. Another 16p11+/− mouse model demonstrates an increase in the relative volume of the nucleus ambiguus and pallidum, as well as a decrease in dopaminergic cells in cortical layers V and VI. Although the relative brain volume alterations in certain animal models may not match those reported in people with CNV in this chromosomal region, their usage can reveal cellular pathways. The relative volume changes in animals modeled with 16p11.2 repeats (DP/+) are the inverse of those seen in DF/+ mice. Although certain animal models cannot replicate the abnormalities reported in CNV patients in this chromosomal region, employing these models can show the molecular pathways that cause brain volume changes.

22q11.2 deletion syndrome: Subjects carrying the 22q11.2 deletion are more likely to develop DiGeorge syndrome and are predisposed to various neuropsychiatric diseases [94]. A meta-analysis estimated the prevalence of ASD in deletion bearers to be 11%. When individuals with ASD having the 22q11 deletion were comparing with controls, the right amygdala volume was increased. More medium-sized multispiny neurons and interneurons clustered in the caudate nucleus in the subcortical white matter. In hippocampal CA1, dendritic complexity, spine density and PSD length are not affected in the Df1/+ mouse model with 22q11.2 deletion. In vitro cultures of hippocampal neurons from DfA++ animals revealed lower numbers of mushroom spines, diminished spine length, fewer glutamatergic synapses, and reduced presynaptic vesicle density. The numbers of cells in layers II and V were decreased in DfA++/− animals, as were inhibitory neurons in layer V. The length and intricacy of the basal dendrites were reduced.

### 2.3. Idiopathic Animal Model

ASD is a neurological illness produced by a combination of circumstances, and mutations in single genes do not adequately duplicate all the clinical symptoms of ASD. Inbred strains of mice and rats displayed substantial and well-replicated ASD-related social impairments and repetitive behaviors in recent years. It is thought to be a model of idiopathic autism.

BTBR-T+ tfl/J (BTBR): In terms of core ASD behavioral traits, BTBR mice are the most fully studied and are the most frequently reproduced inbred breed [95]. The lack of the corpus callosum and significant shrinking of the hippocampal confluence characterize BTBR animals [57]. When BTBR mice were compared to controls, their brain volume was lowered [59]. These findings are consistent with an increase in gray matter volume with time in ASD and are strongly correlated with the seriousness of symptoms. Other notable differences between BTBR mice and control mice included more neuronal expression of 5-hydroxytryptamine in the median and dorsal caudal spine and fewer axonal terminals in hippocampal CA1 [58], but increased postnatal turnover of excitatory synapses in the prefrontal cortex.

BTBR mice performance is connected with genetic alterations in the brain, including brain-derived neurotrophic factor (BDNF) and synaptophysin. Steinmetz et al. showed that injecting insulin-like growth factor 2 prior in BTBR mice before behavioral testing can reverse abnormal behavior and memory deficits [96]. Social interaction and communication in BTBR mice were increased by administering beta carotene according to Avraham et al. [97]. Silverman et al. reported that by injecting BTBR mice with GRN-529, a modulator selectively metabolizing glutamate receptor subtype 5, excitatory neurotransmission was manipulated, and ASD-like symptoms were improved. As mentioned above, immune system dysfunction was thought to be a factor contributing to ASD-like behavior in BTBR mice. Indeed, Schwarzer discovered that irradiating BTBR mice, achieving bone marrow ablation, then injecting bone marrow cells from normal C57BL mice improved mice’s social competence. Studies that proved transplanting bone marrow cells from BTBR mice into C57BL mice and observed that C57BL mice exhibited an increased number of repetitive grooming activities, thus demonstrating the importance of the immune system in social behavior. The advantage of these animal models above is that they allow for molecular and pathological studies of specific brain changes, along with gene editing in an attempt to reverse these ASD-like behavioral changes [98].

### 2.4. Environmental Models

Models of environmentally induced ASD-like behaviors attempt to treat offspring directly by affecting the mother or early after birth. Generally divided into infectious/inflammatory means and specific chemicals (valproic acid) promoting autism-like behavioral traits.

Specific chemicals (valproic acid) exposure: VPA is a short-chain fatty acid used extensively as an antiepileptic and mood stabilizer [99]. Clinical studies have shown an increased risk of numerous neural tube defects, extracerebral malformations, developmental delays, cognitive impairment and autism when VPA is taken during pregnancy [100]. Fetal valproic acid syndrome (FVS) develops from prenatal exposure to VPA, and children with FVS display a significantly increased incidence of developmental problems, decreased verbal intelligence, and often comorbid communication problems associated with ASD. Intriguingly, rodents exposed to VPA before birth exhibited deficient behavior comparable to that of autistic individuals. Thus, the VPA rodent model has been widely used as a common model for studying the neurobiology of autism and screening new drugs [101].

There is now substantial evidence that maternal challenge with VPA in rodents is a favorable animal model for autism. A possible link between maternal exposure to VPA and offspring ASD was first described by Christianson et al. Later, larger studies confirmed the association between intrauterine exposure to VPA and autism. Based on the Diagnostic and Statistical Manual of Mental Disorders criteria, statistics found that 8.9% of 56 children with prenatal exposure to VPA in the monotherapy study developed autism or Asperger’s syndrome. A large study based on the Danish population revealed a 2-fold increase in the prevalence of ASD among 508 kids prenatally exposed to VPA. Prenatal exposure to VPA in rodents is associated with behavioral and neuroanatomical deficits, including reduced social interactions, greater repetitive behaviors, and more anxiety, accompanied by a reduction in the number of cerebellar Purkinje cells, nucleus damage, and cortical synaptic changes like those observed in ASD humans.

Acute higher doses of VPA exposure resulted in decreased brain weight, increased cortical layer thickness, cell density in the prefrontal and somatosensory cortices, and hippocampus. On the other hand, lower doses had no effect on brain weight, but affected cortical thickness and neocortical cell density. VPA exposure leads to thinning of the early prefrontal cortex (PFC), basolateral amygdala, and hippocampal CA1 [102,103]. VPA exposure leads to a decrease in microalbumin-expressing interneurons in the parietal and occipital cortices and a decrease in the number of motor neurons in some brainstem motor nuclei [64]. VPA-treated rats showed an increase in microglia in the medial PFC and astrocytes in the hippocampus [104].

Current studies have reported that using antioxidants such as astaxanthin, piperine, and green tea extract are effective in preventing VPA-induced ASD-like behavior in rats and mice [105]. The polyunsaturated fatty acids α linoleic acid (ALA) or γ linoleic acid (GLA) attenuate neurobehavioral changes in VPA rat offspring by reducing oxidative stress marker concentrations [106]. A recent study found that VPA rat offspring, injected with guanfacine (an endogenous NMDA receptor antagonist) at one-half hour prior to behavioral testing, alleviated ASD-like symptoms. Interestingly, repetitive behaviors in VPA rat offspring were prevented by low-dose injections of donepezil [62]. A current study found that repetitive behaviors were significantly reduced and social skills improved using human adipose-derived stem cells injected into VPA rat offspring ventricles [107]. However, these drugs must be administered continuously to be effective. There are no data to support successful prevention of VPA-induced neurobehavioral impairment in humans. 

Maternal immune activation: Maternal immune activation during pregnancy is highly associated with ASD incidence in offspring [108]. Studies have showed that maternal immune activation leads to alterations in levels of multiple interleukin-like factors in the fetal brain, accompanied by morphological abnormalities in different brain regions [65]. Maternal mid-pregnancy injection with endotoxin and polysaccharide polycyclic acid induced autism-like behaviors in offspring. Viral influenza infection in pregnant mice at mid-gestation resulted in decreased social competence in the offspring. Infection with Borna virus in rats leads to increased stereotypic behaviors associated with autism in offspring. Pioglitazone, an agonist of peroxisome proliferator-activated receptor gamma with anti-inflammatory effects, has been shown to attenuate autistic-like behavioral changes in the offspring of endotoxin-treated rats [109]. These models can be utilized to explore neurological and behavioral changes along with the molecular and biochemical mechanisms involved in neurological pathology, particularly in the hippocampus and cerebral cortex. The majority of these animal models assess inflammatory processes rather than specific viruses.Animal models of genetic mutations in ASD reveal differential effects of genetic variation in individual genes on ASD-related phenotypes. But ASD is heterogeneous, and it is difficult for a single transgenic model to recapitulate all its symptoms. At the same time, transgenic models are susceptible to the influence of genetic background. CNV animal models have high genetic penetrance and a strong association with ASD manifestations. But CNVs are less common in the general patient population. Idiopathic animal models mimic many of the aberrations found in the ASD population. But the behavior of this strain of mice must be compared with that of other unrelated mice [110]. The rodent with VPA is an important model of autism and may be more representative of many cases of idiopathic autism due to environmental/exogenous causes than autism models carrying mutations in a single associated gene. However, most people with autism are not exposed to the drug before birth. Therefore, the validity of the VPA rodent model may be limited to cases of autism resulting from exposure to drugs that act as histone deacetylase (HDAC) inhibitors.

## 3. Importance of Multidisciplinary Assessment of Animal Models of Autism

At present, clinical ASD is mainly diagnosed through the basis of behavioral disorders and no specific neurodiagnostic markers are available. Therefore, tools to assess the development of ASD-like behaviors must be created. Currently established ASD rodents assess ASD-like behaviors in humans primarily through the following behavioral tests. In order to explore potential tools that may engage in the neurobiological mechanisms of human ASD, a multi-dimensional approach is proposed to study animal models of ASD, combining core behaviors, neuroimaging, neuropathology, and neurochemistry in animal models.

### 3.1. ASD Core Behavioral Testing

Core behaviors in ASD mainly show behavioral cognitive deficits of social behavior impairment, repetitive behaviors, and cognitive rigidity. It is mainly assessed by ultrasonic vocalization test, open-ended test, social novelty preference test, maze experiment, and novelty recognition test.

Ultrasonic vocalization in mice may be a valuable marker to differentiate between the control and ASD models [111]. Autism-like disorders can be detected in interactions between offspring and their mothers. The number of ultrasonic vocalizations decreased after separation of prenatal VPA-exposed pups from their mothers in isolation testing, while the duration of vocalization events consistently increased [112]. Where calls had a lower amplitude, there were more flat calls, a change in the number of complex and downward calls, less variety in call types, and fewer 2-syllable calls [113,114]. Instead, offspring injected with VPA between gestation days 11 and 13 exhibited increased call frequency [115]. Other flaws included lower call amplitude, complicated and downward call number alterations [113]. Although contradictory to earlier findings in the literature, it may be attributable to the divergent dose ranges of VPA employed in various experiments.

Open-field tests are used to evaluate changes in gross locomotor activity in rodent performance levels. The test is commonly applied in classical experiments to detect core symptoms of an ASD diagnosis including stereotyped motor behavior, repetitive self-modification, and restriction of exploration activities. The open-ended test is strongly associated with social behavior and anxiety in mice and rats with autism [116].

Three-chamber social interaction tests are used to measure social competence and preference for social novelty by calculating the ratio of time spent on novel social stimuli to time spent on familiar social stimuli. In rats and mice exposed to VPA, the three-compartment social approach revealed deficits in social interaction [117]. Markram discovered that VPA- exposed rats and mice exhibited reduced play activity and exploration of heterotypic adventures, which lends more credence to the effect of VPA on sociality.

The T-maze test is used to assess memory and cognitive abilities in rodents. The animal’s capacity to spontaneously alternate is examined using a closed apparatus, and is primarily affected by hippocampal dependent function [118]. Water T-maze serves to measure reversal learning, cognitive rigidity, and repeated behavior. The animal must suppress the first learning reaction and learn a new place on the platform [119]. Three-arm maze is used to evaluate short-term memory, measuring mainly hippocampal function in mice and rats [120].

### 3.2. Neuropathology

Neuropathological studies can evaluate subtle features affecting ASD patients’ brains such as neuronal differentiation migration, morphology and spatial distribution. Numerous neuropathological examinations have been performed in ASD, and here we provide an update of these results.

Neuron size, number, and density: Early autopsy studies compared the number of neurons and neuroglia in various regions of the cerebral cortex of autistic patients to age- and gender-matched controls. Autistic brains had lower neuroglial/neuronal ratios across the board, but no significant differences in cell density were detected [121]. Another study discovered 79% more neurons in the DL-PFC and 29% more neurons in the M-PFC in 7 male autistic children than in 6 controls [122]. The autistic group’s brain weight was slightly higher than average, indicating a pathological increase in neuron number. A stereological study [123] estimated neuronal volumes in the cortical structures, hippocampus, arches, cerebellum, and brainstem of 14 autistic individuals. Neuronal volumes were observed to be reduced in the locations studied. Significant deficits in 14 subregions were detected in four autistic patients aged 4–8 years, but volume deficits were found in only three or four of the 16 examined regions in six subjects aged 11–23 years and four subjects aged 36 years. Purkinje cells and neurons in the claustrum have consistently decreased neuronal volumes throughout their lifespan. However, the developmental trajectory of neuronal volume changes revealed an increase in neuronal volume in both adolescents and adults with autism, as well as a decrease in neuronal size in most regions in older controls, indicating an abnormal neuronal growth trajectory.

Neuronal migration impairment: It has been proposed that cerebral cortical abnormalities found on magnetic resonance imaging examination of autistic individuals are caused by a malfunction in neuron migration to the cerebral cortex during the first 6 months of gestation. Other signs of cortical dysgenesis identified in autism patients include thicker cortex, high neuronal density, minicolumnar changes, the presence of neurons at the molecular layer, abnormal laminar patterns, weak grey-white matter borders, and ectopic grey matter [124]. The discovery of lower levels of Reelin in post-mortem cerebellum tissue from autistic patients lends credence to the idea of disrupted neuronal migration in autism. Reelin expression was demonstrated to be decreased in the cerebral cortex of pregnant mice offspring exposed to human influenza virus in pregnancy [125].

Other neuropathologies: Research found disturbances in cortical cell patterning in the superior temporal gyrus, dorsolateral frontal lobes, and dorsal parietal lobes, including dysplasias, related lamination disturbances, and a vaguely less defined gray/white matter border in 8 ASD participants. The most reliable indicator of an affected area was a lack of expression of excitatory cortical neuron markers [126]. These patches of aberrant laminar cytoarchitecture and cortical disarray were observed in neurons but not in glia. However, there were significant differences in which cell types and layers were most affected by the pathological features. In ASD patients, a dramatic reduction was observed in pyramidal neuron size in the inferior frontal cortex suggesting that long distance communication is hindered. This was confirmed by neuropathology [127]. Meanwhile, hypoactivation of the syrinx gyrus was observed in the temporal cortex of ASD, which may be related to reduced mitochondrial energy metabolism. The subventricular zone of the lateral ventricles is one of two neurogenic niches in the brain that are required for neural proliferation, migration, and differentiation throughout both prenatal and postnatal development. Early analysis in ASD patients with the amygdala showed reduced volume and higher neuronal density in the medial, central and cortical nuclei, whereas the most recent quantification in ASD patients showed a significant reduction in the number of neurons in the amygdala as a whole or in the lateral nucleus of the amygdala [128]. The reduction in the number of neurons might be due to less neurons formed during the developmental process or could be caused by abnormal degeneration of cells that occurs after normal early development loss.

### 3.3. Neuroimaging

In the past decade, in vivo MRI research has contributed many useful insights into the neural basis of ASD. Human neuroimaging research may contribute to biomarker development for ASD and other neurodevelopmental disorders, as well as novel approaches to diagnosis and treatment.

A growing number of neuroimaging studies support early atypical brain development and widespread alterations in ASD neurological connections. Normal brain development is reliant on both cellular and synaptic growth, as well as the properly timed trimming of neurons and synapses [129]. This balance appears to be compromised among some children with autism. A recent neuroimaging study addressing 6 months of age at-risk infants with ASD showed that the aforementioned children exhibit abnormal connectivity in brain trajectories. According to multiple studies, the extent of atypical connectivity at 6 months of age relates to future symptom severity [130,131]. Children with ASD exhibit a sustained expansion of cortical surface area from auditory and visual processing sensory areas beginning at 6 months to 12 months of age, with overdevelopment at 12 months to 24 months of age [132]. Similarly, the overgrowth observed in individuals with ASD may not represent new neurodevelopment because neuronal cells may not be apoptotic and pruned early in development due to neuronal overgrowth. Children with autism continue to have larger brain sizes than their counterparts from the age of 2 to 4 years. By school age, brain development has slowed and the brain volume of normally growing children is catching up to the brain volume of children with autism. Studies of connectivity have revealed persistent impairments in the way brain regions are connected throughout childhood, adolescence, and maturation. A major pooled study of functional MRI resting-state data from ASD patients compared to age-matched normal controls showed widespread low connectivity in distant cortico-cortical and hemispheric projections. In contrast, subcortical regions exhibited local hyperconnectivity [133]. Overall, these findings indicate that higher brain activity requiring communication between brain regions are suppressed to the benefit of local circuits that may be hyperactive and difficult to interrupt.

Neuroimaging studies are advancing us in understanding the biology of ASD, however, there is no evidence to support that routine neuroimaging can confirm a diagnosis of ASD. Before neuroimaging may be considered for clinical application, further research is needed to better understand and standardize the developmental trajectory of the brain in ASD, which could eventually be used to detect children at risk of developing ASD before overt symptoms appear.

### 3.4. Neurochemistry

From a neurochemical perspective, brain structures and neural circuit activity are regulated by a combination of neurotransmitters. Changes in neurotransmitter concentrations and dynamics can affect neuron-related functions [134]. Growing evidence suggests disturbances in the neurotransmitter system may be associated with ASD, including mainly GABA, glutamate, serotonin, dopamine, and N-acetyl aspartate, among other agents. 

Gamma aminobutyric acid (GABA), derived from glutamate by the action of glutamic acid decarboxylase, is the most frequent excitatory neurotransmitter in the developing brain and has a complex link to neuronal excitability [135,136,137,138]. Alterations in the gabaminergic and glutaminergic systems can disrupt the excitatory/inhibitory balance, which is a possible causative factor in autistic development. Elevated excitatory/inhibitory balance impairs information processing and causes social-behavioral dysfunction. In mouse models with mutations in SHANK3 and the glial-neuropilin complex, glutamate concentrations in the striatum were shown to be reduced [138]. Furthermore, in magnetic resonance spectroscopy tests, reduced GABA was observed in participants in motor, visual, auditory, and somatosensory regions, as well as in the left hemisphere lateral fissure region, resulting in aberrant information processing [139]. When compared to controls, kids with autism had markedly elevated plasma GABA and glutamate/glutamine ratios, but significantly lower plasma glutamine levels and glutamate/GABA ratios [140]. MECP2 mutant mice change synaptic physiology by decreasing glutamic acid decarboxylase-1 and -2 levels and GABA immunoreactivity, resulting in GABA dysfunction and various autism-like Rett syndrome characteristics. Several studies have highlighted links with GABA receptor single nucleotide polymorphisms [141,142].

*Glutamate* is the major excitatory neurotransmitter in the mammalian cerebral cortex. N-methyl-D-aspartate receptors (NMDARs), α-amino-3-hydroxy-5-methyl-4-isoxazolepropionic acid receptors (AMPARs), and metabotropic glutamate receptors (mGluRs) are the three main categories of glutamate receptors [143]. NMDARs and AMPARs are thought to be associated with ASD [144]. It has been shown that overexpressing NMDA receptor subunits in rodent models of autism enhances synaptic currents mediated by NMDA receptors, thereby enhancing postsynaptic plasticity [145]. Additionally, modifications to the AMPA Receptor 2 (GluA2) subunit can profoundly affect neuronal excitability, which is associated with neuropsychiatric disorders including mental retardation and Rett syndrome. Mouse models of autism containing Cyclin-dependent kinase like-5 deficiency showed significant reductions in GluA2 in the hippocampus [146]. Recent studies have reported that the cerebellum is associated with autism spectrum disorders [147]. Interestingly, it was demonstrated for the first time that the cerebellar granule layer was altered in the islet brain-2 (IB2) KO mouse model and triggered autistic symptoms and severe delayed motor deficits. The IB2 KO mouse model has high activity and plasticity of NMDA receptors, which determines an increased excitatory/inhibitory balance and enhanced long-term potentiation of mossy fibers and granule cells [148]. Also, early correction of NMDAR dysfunction in a mouse model showed dramatic improvements in autistic-like behavior [149]. Mutations in synapse-formation and -maintenance genes as well as protein-targeting genes, have been linked to development of autistic traits and glutamatergic dysfunction [150].

Serotonin (5-hydroxytryptamine, 5-HT) is a monoamine neurotransmitter that influences a variety of brain activities including memory and learning capacity [151]. moreover, acting as a sleep and mood regulator [152]. Serotonin transporter protein or serotonin levels are higher in children with autism and animal models than in controls. Studies have indicated that whole-brain 5-HT synthesis is reduced during childhood in children with autism, but gradually increases between the ages of 2 and 15 years, reaching 1.5 times the normal adult value [153,154]. Polymorphisms in the serotonin transporter protein gene (SLC6A4), which encodes platelet and neuronal transport of 5-HT, have been associated with autism. Higher 5-HT levels found in ASD are abundant in children with the SLC6A4 polymorphism [155]. Obviously, valuable animal evidence suggests that embryos developing in SLC6A4+/− mothers are less resistant to prenatal stress, which increases the risk of offspring developing ASD-like traits [156]. Multiple studies have examined platelet hyperosmolarity in ASD subjects, with a mean increase of 20% to 50%. Intriguingly, this increase appears to be unique to autism, as it has not been observed in intellectual disability or other neuropsychiatric disorders.

Dopamine, in addition to controlling locomotion, influences social cognitive and behavioral characteristics via the central cortical circuit [157]. Several investigations have discovered that ASD is connected to dopamine dysfunction [158,159]. According to research, disfunction of mesocorticolimbic circuit causes social impairment in autism, whereas dysfunction of nigrostriatal circuit results in stereotyped behavior [160]. Drug-induced nigrostriatal pathway dysfunction produced stereotypic behavior in mice [161]. Indeed, D1 dopaminergic receptor antagonists were given and these behaviors were reduced. A recent study supports the idea that mesocortical brain circuit can influence social behavior through bidirectional control of dopaminergic projections from the ventral tegmental area to the nucleus accumbens. Optogenetic stimulation of neurons in the dopaminergic ventral tegmental region activates D1 receptors, increasing the amount of time the animal spends on social interactions [162]. Genetic research has demonstrated that autism is linked to polymorphisms in several genes related to the dopaminergic pathway, such as the dopamine receptors DR3 and DR4, or the dopamine transporter protein (DAT) [163]. One recent study concerning a mouse model emphasized the mutation in DAT that triggers abnormal dopamine efflux, leading to an autism-like behavioral phenotype [164].

Acetylcholine is a neurotransmitter and neuromodulator in the central nervous system that is the major neurotransmitter of motor neurons and the parasympathetic nervous system at the neuromuscular junction [165]. Abnormalities in the cholinergic system induce ASD [166]. Current ASD autopsy has revealed substantial decreases in nicotinic subtype acetylcholine receptors (nAChRs) in brain tissues from the parietal and frontal cortex [167]. Another research found a decrease in α4 nAChRs in the cerebellum, considering that it may be related to the loss of Purkinje cells and a compensatory increase in α7 nAChRs. Several investigations on ASD animal models have revealed that nAChRs have a role in controlling social and repetitive behaviors [168]. α4 nAChR subunit knockout and β2 nAChR subunit knockout mice exhibit increased anxiety and abnormal sleep patterns. α7 nAChR receptors are abundantly expressed in the hippocampus and frontal cortex [167], and activation of this receptor has a cognitive-promoting impact in animal models [169]. Furthermore, choline supplementation during pregnancy enhances the fetal brain response to maternal immunological stimulation and avoids certain caused behavioral abnormalities in the offspring.

## 4. Biological Mechanisms and Neural Circuitry of ASD

In general, it is believed that associated gene variants in ASD patients and animal models are likely focused on similar molecular or cellular pathways. ASD is closely associated with gene transcription, mRNA, and functionally significant non-coding mRNA, altered synaptic signaling pathways, abnormal epigenetic post-translational modifications, and immune and inflammatory aspects. Preliminary evidence has been acquired at the animal level. However, epigenetic networks are intricate and often interact in a cascade fashion. Abnormalities in transcription and translation exacerbate abnormal neuronal function in ASD, further affecting synaptic transmission and plasticity, and dysregulation of the gut flora affects peripheral immune response function also influences brain dysfunction. Affects the formation and activity of neural circuits; conversely, modified neural activity can influence further transcription factors or chromatin remodeling by transmitting trigger signals and initiating action potentials for specific transcriptional programs. Future research is needed to further elucidate the underlying pathogenesis of ASD in depth.

### 4.1. Activity-Dependent Gene Transcription

MECP2 is a transcriptional repressor whose deletion increases global transcriptional levels and alters chromatin structure [170]. MeCP2 is involved in neuronal activity by phosphorylating and dissociating nuclear receptor co-chaperones at S86, S274, and T308. Notably, MECP2 binds to chromatin and transcriptional activators at activated target promoters to activate gene expression, implying that MECP2 may act as both a transcriptional activator and a repressor. Activity-dependent neuroprotective proteins directly encode transcription factors that bind and regulate the DAT transcriptional activator ZFP161 and the transcriptional repressor FMR1 [171]. Myocyte enhancer factor 2 (MEF2) is an active regulatory transcription factor that regulates ASD-related genes such as Protocadherin 10, UBE3A, and BDNF. Mutations in the gene encoding UBE3A occur on chromosome 15q11 in patients with Angelman syndrome and some ASD patients [172]. T-box brain 1 is a neuron-specific transcription factor that is necessary for activity-dependent Grin2b expression, and the loss of one copy alters the expression of netrin G1, contactin-2, and Cadherin-8 (CDH8) [173]. 

### 4.2. mRNA Translation and Non-Coding RNA

Fragile X syndrome mental retardation protein (FMRP) is an mRNA binding protein that is abundant in the brain and regulates a large number of mRNAs. FMRP was found to selectively bind 4% of mRNAs in the mammalian brain [174,175]. The most characteristic motifs in FMRP are those that interact with RNA: two hnRNP-K-homology (KH) domains and an arginine-glycine-glycine (RGG box) [176]. In vitro, the RGG box identifies the STEM-G-Quartet loop in RNA, and similar G-Quartet structures have been discovered in various FMRPs. MAP1B, a microtubule-associated protein critical for axon formation, is encoded by FMRP ligand mRNAs with G-quartets. It has been shown that FMRP can bind to structured G-rich regions, which include typical G-quartets. Cytoplasmic polyadenylation element binding proteins 1–4 (CPEB1–4) are RNA binding proteins that inhibit or activate translation of mRNAs with CPE sequences in their 3’ untranslated regions (UTRs) by inducing cytoplasmic shortening or lengthening of their poly(A) tails [177,178]. CPEBs are involved in learning and memory by regulating embryonic development and synaptic plasticity [179]. In the brains of individuals with idiopathic ASD, CPEB4 protein levels are reduced but transcript levels are elevated, disrupting the auto amplifying loop that regulates CPEB4 levels [178], consistent with CPEB4 binding to its own transcripts. CPEB1 deletion rescues the fragile X-like phenotype of FMR1 knockout (KO) mice.

Most of the existing research into whole-genome linkage has focused on protein-coding regions, ignoring noncoding RNAs, which are considered nonclassical epigenetic pathways because they primarily target transcripts and rarely interact directly with DNA [180]. Post-transcriptional regulation of noncoding RNAs includes short noncoding RNAs (miRNAs) and long-stranded noncoding RNAs (lncRNAs). miRNAs regulate the expression of most genes by blocking protein synthesis or increasing mRNA degradation at the post-transcriptional level [181]. Preliminary assessments have shown that 28 miRNAs are markedly changed in postmortem cerebellar cortical tissue of ASD patients. Intriguingly, differential expression of miRNAs predicted targets and identified additional genes related to neurobiology, cell cycle, and cell signaling. In animal studies, knockout miR-137 heterozygous mice exhibited repetitive and social behavior deficits [182]. The miRNA expression profiles in the cerebellums of MECP2 knockout mice revealed downregulation of miRNA subsets [183], consistent with the finding that miR-132 targets MECP2 and BDNF in vitro and is downregulated in the cortex of MECP2 knockout mice. Thus, regulatory loops including BDNF, miR-132, and MECP2 may be involved in ASD. Furthermore, considering miRNAs can regulate gene activity without integrating into the host genome, targeting miRNAs is a promising strategy for ASD treatment [184].

LncRNAs have been demonstrated at high levels of expression in the central nervous system (CNS). CNS cells have high levels of lncRNA expression, with 5458 out of a total of 9747 lncRNA transcripts detected. lncRNAs can be transcriptionally regulated by isolating splicing factors or by regulating the distribution and phosphorylation of splicing factors in splice sites [185]. Recent research has identified that metastasis-associated lung adenocarcinoma transcript 1 (MALAT1) affects the expression of neuronal synaptogenesis genes by modulating serine/arginine splicing factors [186,187]. LncRNAs are capable of regulating RNA translocation, translation, and degradation as well [188]. To date, two studies have examined changes in regulatory lncRNAs in ASD brain tissue [189]. The researchers discovered that the expression of 222 lncRNAs differed between ASD patients and controls. The prefrontal cortex and cerebellum were related to 82 and 143 of the 222 lncRNAs, respectively. The finding is important in the context of autistic brain imaging studies, which also found that the number of lncRNAs differentially expressed in control brains was much greater than the number of lncRNAs differentially expressed in autistic brains (1375 lncRNAs and 236 lncRNAs, respectively) indicating that there are fewer specialized regions in the brains of patients with autism than in the brains of healthy subjects [190,191]. Parikshak and colleagues then conducted a larger sample study to examine lncRNA, splicing, and regional gene expression patterns in autism through postmortem genome-wide transcriptome analysis [189]. They examined 251 postmortem frontal and cerebellar regions enriched in autism risk genes in the postmortem frontal and temporal cortices and cerebellum. The results suggest that lncRNA dysregulation is an integral part of the ASD transcriptome signature.

### 4.3. Synaptic Signaling Pathway

*Wnt signaling pathway.* The Wnt signaling pathway has long been associated with neuronal overgrowth, and its changes are considered to be pleiotropic in the etiology of autism [192]. Molecular, biochemical, electrophysiological, and behavioral abnormalities related to an autism-like phenotype were observed in various Wnt signaling pathway-related knockout mice models [193]. Wnt signaling has two main pathways: (1) β-linked protein-dependent stable “canonical” signaling and (2) β-linked protein non-dependent “non-canonical” signaling. Significantly, many of the key protein signaling pathways in both are localized at the synapse and have a crucial role in synaptic maturation [194].

Typical Wnt signaling indirectly acts on β-linked proteins to enhance their stability and translocate them out of the cell surface to the nucleus, thus transducing extracellular signals with downstream transcriptional mechanisms regulating nuclear gene expression. Several ASD-related genetic mutations are connected to dysregulation of the traditional Wnt/-linked protein pathway via the interaction of the CHD8 and beta-catenin [9]. On the one hand, CDH8 is a negative regulator of autism [195], and CDH8 involves in the classical Wnt signaling pathway by directly binding to β-catenin or being recruited to the promoter regions of β-catenin-responsive genes [193]. Free cytoplasmic β-catenin is phosphorylated by GSK3β resulting in degradation by the proteasome [192]. Several studies have also found that phosphatase and tensin homolog (PTEN) are involved in Wnt signaling by regulating normal brain growth together with β-catenin [192]. PTEN haploinsufficient mice have brain overgrowth and enhanced β-linked protein signaling, emphasizing the role of PTEN and β-catenin signaling in regulating a role in normal brain growth

PI3K-AKT/mTOR signaling pathway. PI3K-AKT/mTOR signaling pathway is highly associated with autism and various neurodegenerative diseases [196]. The PI3K-AKT/mTOR signaling pathway is primarily regulated by phosphatidylinositol 3 (PI3K) and its downstream serine/threonine protein B (PKB; also known as AKT), as well as the mammalian/mechanical target of rapamycin (mTOR) [197,198]. The PI3K-AKT/mTOR signaling pathway is stimulated by receptor tyrosine kinase (RTK) and cytokine receptor activation [199,200]. The PI3K-AKT/mTOR signaling pathway is primarily engaged in synaptogenesis, corticogenesis, and related neuronal regulatory processes [201].

Studies have identified a mutated PI3K-AKT-mTOR pathway in nearly 50% of kids with brain malformations and delayed development/autism [202]. The AKT/mTOR pathway is a biological substrate for autism, and mutations in the AKT/mTOR gene cascade lead to an increase in autism-like behaviors by regulating translation to dendritic spines. Further studies have found that decreased mTOR affects advanced cognitive and behavioral cortical circuits leading to an autistic phenotype [203].

PTEN activation contributes to axon elongation and proper localization. PTEN inhibition stimulates neurite growth [204,205]. When the neurite reaches the target region, the growth cone collapses and recruits PTEN back to the surrounding region, where it mediates collapse in response to chemotactic agents. In the CNS, mTOR is involved in synaptic plasticity, cell growth, migration, neuronal development, memory storage, and protein synthesis [206,207]. Aberrant expression of PI3K/AKT in neurons results in elevated levels of reactive oxygen species, membrane depolarization, mitochondrial instability, neuronal apoptosis, reduced oxidative phosphorylation, and ATP production [208]. The non-phosphorylated form of EIF4E protein-binding protein 2 (4E-BP2) binds to eIF4E to interfere with the formation of the eIF4F complex to block translation and mTORC1 release. Importantly, eIF4E transgenic mice exhibit repetitive and stereotyped behaviors, abnormal social interactions or cognition, as well as a persistent pattern of ASD-like behavioral impairment [209].

ERK/MAPK signaling. The ERK/MAPK pathway is intrinsically linked to autism spectrum disorders and other disorders characterized by mental retardation. ERK is critical in brain development and synaptic plasticity. ERK is activated when Ca2^+^ transients are increased. The ERK activity is changed by mutations in ERK/MAPK pathway elements, leading to a group of classic human syndromes called “Rasopathies” [210,211,212].

In the developing cerebral cortex, neuronal responding to neurotransmitters and RTK ligands is dependent on the ERK/MAPK pathway [213]. Previous experimental results found that damaged ERK in 16p11.2del mice resulted in brain volume reduction and disrupted cortical cell structure in 16p11.2del mice. Inhibition of the Ras-ERK pathway partially ameliorated the abnormal behavior of 16p11.2-deficient mice. RB1 and RB3 are two novel cell-permeable peptides that significantly suppress Ras-ERK signaling during development to rescue morphological damage in a severe Rasopathies mouse model [214,215].

**Figure 2:**
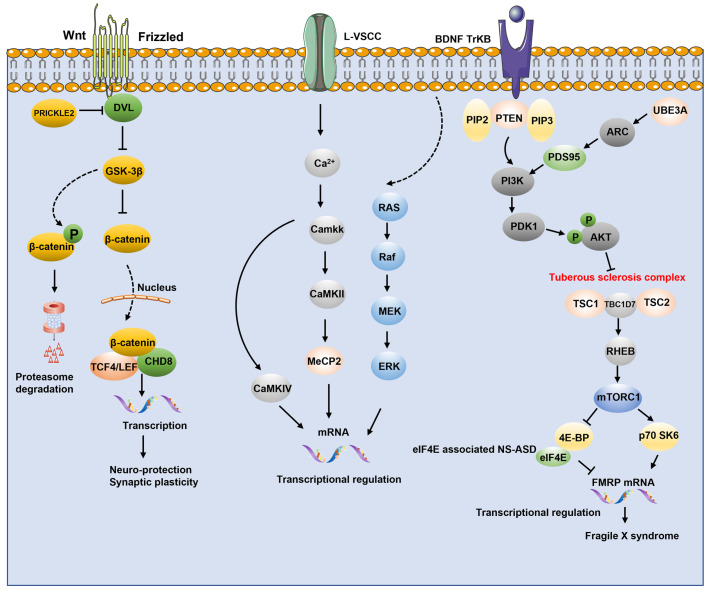
The Synaptic signaling pathway are mechanistically targeted. Wnt pathway activation of cytoplasmic β-catenin is degraded by the proteasome after phosphorylation by GSK-3β. When GSK-3β fails to phosphorylate β-catenin, cytoplasmic β-catenin accumulates in the nucleus and binds to the TCF/LEF complex and CHD8 factor to activate transcription of target genes and enhances synaptic plasticity and neuronal protection. Both ERK/MAPK and PI3K-AKT-mTOR signaling pathways can be activated in response to TrKB stimulation Activation of L-type voltage-sensitive calcium channels (LVSCCs) triggers calcium inward flow and induces calcium-dependent signaling molecules and the Ras/ERK pathway, which are involved in transcriptional regulation. Mutations in proteins involved in translational regulation include PTEN, MECP2, UBE3A and TSC1/TSC2. These genes are marked in red.

Conditional deletion of the upstream kinase Map2k1/2 causes severe disruption of neurons in layer V, as well as a significant reduction in CTIP2 large neurons and a significant impairment of distant axonal extension of corticospinal projection neurons in layer V during early development. CST neurons in layer V are also affected by ERK/MAPK signaling, resulting in decreased axonal elongation and increased axonal branching. Furthermore, ERK/MAPK signaling inhibition affects excitatory and inhibitory neurotransmission in pyramidal neurons and changes intrinsic excitability in layers II/III and V [213]. Layer Vcorticospinal neurons are highly susceptible to loss of ERK/MAPK signaling in the neonatal period. The expression of plasticity-related proteins and intrinsic excitability within neurons depends on ERK/MAPK signaling in multiple cortical layer [213] (Figure 2).

### 4.4. Epigenetic Post-Translational Modifications

*Methylation modification.* DNA methylation performs an important role in maintaining genomic stability and regulating cellular function. A variety of functions have been demonstrated for DNA methylation in transcriptional regulation, including silencing repetitive elements, changing transcription factor binding sites and chromatin accessibility, and directing the use of alternative promoters and splicing [216].

**Figure 3:**
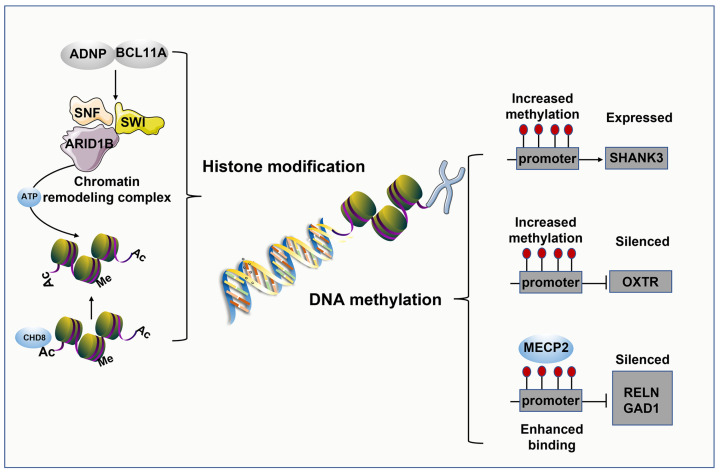
Methylation and acetylation modifications associated with ASD. DNA methylation frequently results in transcriptional repression or even gene silence in impacted genes. MECP2 binds to methylated CpG sites in gene promoters and binds to chromatin silencing complexes, inhibiting gene expression; protein modifications and chromatin remodeling in the acetylation group contribute to transcriptional activation or inactivation and chromatin packaging.

Initially, studies into ASD-related methylation were mainly focused at the genetic level. MBD of MECP2 acts as a reader for several forms of DNA methylation, interacts with multiple neurodevelopmentally relevant transcription factors, and disrupts critical links between cellular functions by regulating chromatin-structure-driven DNA methylation [217]. In ASD patients, an enhanced combination of MECP2 with GAD1 and GAD2 promoters has been detected in the cerebellum and frontal cortex, resulting in reduced RELN and mRNA expression. Oxytocin receptor (OXTR) is a strongly conserved G-protein-coupled receptor. OXTR mRNA expression is affected by promoter methylation, and elevated levels of methylation are correlated with ASD [218,219]. This is consistent with enhanced OXTR promoter methylation found in adults with ASD. Thus, identification of highly specific DNA methylation could contribute to the prediction of transcriptional regulation in autism [180] (Figure 3 right).

Acetylation modification. Histone acetylation is closely related to synaptic function, neuronal excitability, and immune response genes in ASD [220]. In humans, 18 HDAC enzymes have been identified, divided into the HDAC family and the Sir2 regulatory family. HDAC and histone acetyltransferases cause pleiotropic downstream effects through changes in acetylation levels, inducing cognitive dysfunction [221]. Knockout of the forebrain excitatory neuron HDAC3 resulted in comparatively poor social skills in mature wild-type mice [222]. HDAC2 upregulation in the PFC resulted in lower histone acetylation levels in the SHANK3-deficient mouse model. Acetylation group changes have also been proven to influence social hormone receptors [223]. Inhibition of postnatal HDAC by TSA or sodium butyrate caused enhanced histone acetylation of the oxytocin receptor and the pressor V1a receptor gene promoters in the nucleus ambiguus, resulting in social pair bonding in male and female adult prairie voles [224]. A study demonstrated that PFC histone acetylation was decreased at term in crab-eating monkeys exposed to VPA before birth [225], in contrast to results found in rats following short-term suppression of prenatal HDAC [226], indicating that the long-term consequences of HDAC inhibition during pregnancy may differ from the acute effects. A group-wide association analysis of histone acetylation found that 68% of patients with idiopathic ASD and duplication 15q syndrome had similar patterns of histone H3 acetylation on lysine 27 (H3K27ac), which is assumed to be a marker of active enhancers and promoters. In ASD patients, increased acetylation sites are concentrated in neuronal function areas and decreased acetylation sites in areas associated with immune function, with genes encoding HDACs (HDAC2 and HDAC4) in the frontal and temporal cortices [227] (Figure 3 left).

Phosphorylation modification. Phosphorylation is the most abundant post-translational modification (PTM) of proteins. Its importance is revealed by the space assigned to kinases in the genome. In the human genome, more than 500 kinase genes have been identified [228]. Post-translational modifications of phosphorylation are widely involved in neurological disorders. Current research indicates that phosphorylation is closely related to neurological disorders such as depression, Alzheimer’s disease, and autism [229]. Recent studies on the potential mechanisms of phosphorylation in models of autism are increasing. The GABAA receptors of the β3 subunit (GABAAs) mediate sustained alterations in the inhibitory effects of neurons associated with autism spectrum disorders (ASD). Studies have shown that mutating S408 and S409 of GABAAs to alanine enhances the plasma membrane stability of GABARs to block the phosphorylation-dependent regulation of GABAARs, altering the balance between phase inhibition and tonic inhibition in the dentate gyrus [230,231]. Regulation of PTEN activity and function primarily involves phosphorylation of the PTEN C-terminal cluster of serine and threonine residues. Several phosphorylation sites in the C-terminal tail of the PTNE gene are required for the regulation of PTEN stability and activity. Mice with PTEN deletion exhibited neuronal hypertrophy and morphological changes, and PTEN-effective neurons displayed increased dendritic structures and connectivity changes [232]. FMRP is a target of S6 kinase and PP2A phosphatase. Phosphorylated FMRP prevents translation by binding mRNA, ribosomes, and eIF4E1 [233]. FMRP blocks translation by stimulating the excitatory postsynaptic membranes of pyramidal hippocampal cells on mGluR receptors to control the efficiency of dendritic mRNA translation. Dephosphorylation disrupts the binding of FMRP to its targets, thereby activating mRNA translation [234].

Ubiquitination modification. Ubiquitin-proteasome system (UPS) is the central mechanism of protein degradation and turnover. The process is a reversible enzymatic cascade reaction. The protein is labeled through a series of enzymatic reactions and then degraded by covalently linking the K48 polyubiquitin chain to specific lysine residues of the target protein by E3 ligase. Its degradation is inhibited by restoring the initial state by deubiquitinating enzymes. In neurons, UPS is an essential mechanism for regulating excitatory synaptic activity-dependent protein turnover and structural changes.

PSD scaffolding molecules, SHANK and the guanylate-kinase-associated protein (GKAP) and PSD95-associated families, are the most highly ubiquitinated proteins in the synapse, where protein levels are controlled bidirectionally by synaptic activity [235,236]. SHANK proteins are highly concentrated in the PSD, which interacts with GKAP and PSD-95 family members. Altogether, these scaffolds support the structure of the PSD and coordinate intracellular responses to extracellular and intracellular signal transduction enzymes by binding to multiple membrane receptors, including corticosteroids and CAMKII78. USP8 controls SHANK3 and SHANK1 protein levels through de-ubiquitination, which further comes to control dendritic spine density in neurons. In addition, knockdown of USP8 in neurons blocks synaptic activity and alters SHANK3 protein levels [237]. The UBE3A gene is closely associated with autism development. UBE3A is a HECT family E3 ligase, and UBE3A targets XIAP for ubiquitination and degradation, subsequently increasing the level of cystein 3 activation, leading to microtubule cleavage and eventual contraction and removal of dendritic branching structures [238]. In the UBE3A ASD mouse model, cortical neurons were significantly reduced [239]. Several autism-related genes were found to mediate synaptic elimination via the proteasomal degradation of the synaptic scaffold PSD-95. For example, PSD-95 is ubiquitinated upon MEF2 activation by ubiquitin E3 ligase MDM2 and then combined with Pcdh10, which attaches it to the proteasome for degradation.

SUMOylation modification. SUMOylation is a PTM involved in many cellular signaling pathways. It consists of small ubiquitin-like modifier (SUMO) proteins that bind covalently to specific lysine residues of substrate proteins to exert biological functions [240]. In neurons, SUMOylation is a critical transcription regulator and serves an essential role in synaptic function and neuroprotective responses to severe stress.

SUMOylation regulates widespread neurodevelopmental processes [241,242]. The SUMO system in the developing rat brain was shown to be spatiotemporally regulated, and SUMOylation is regulated by neuronal activity and activation of mGlu5R [243]. SUMOylation also affects various aspects of neurological function, including neurotransmitter release, spinogenesis, and synaptic communication. SUMOylation of Synla at K687 was found to enhance synaptic association with the synaptic vesicle to promote efficient reassembly of synapses and maintain its presynaptic localization after neuronal stimulation. The A548T mutation in Synla damages its own SUMOylation, which may explain the abnormal phenotype of epilepsy and ASD associated with this mutation. FMRP is a substrate of the SUMO pathway in the brain, and FMRP SUMOylation functions through the activation of mGlu5R and is necessary to maintain the shape of mRNA particles in dendrites and to control spine density and maturation [243].

**Figure 4:**
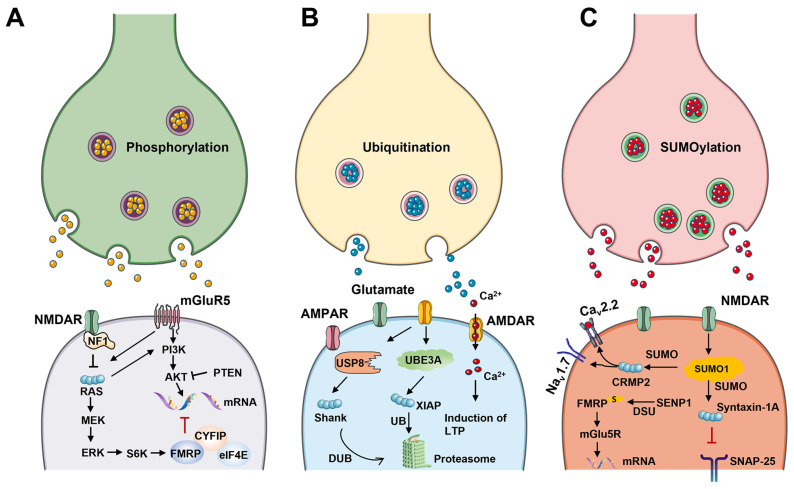
A network of epigenetic post-translational modifications associated with ASD pathophysiology. (**A**) Ras inactivation is generally caused by neurofibrillar proteins encoded by NF1. Neurofibrillary protein mutations in neurofibromatosis 1 cause excessive activation of the Ras/ERK and PI3K signaling pathways, affecting and inhibiting translation of proteins encoded by genes associated with neurodegenerative diseases in autism. (**B**) USP8 is required for synaptic function by stabilizing Shank family proteins to prevent degradation. UBE3A regulates synaptic activity by targeting XIAP ubiquitination. (**C**) FMRP activity-dependent SUMOylation is a critical step in the separation of FMRP from dendritic mRNA particles, which regulates spine elimination and maturation. CRMP2 is a SUMO substrate that reduces Ca2^+^ entry via the presynaptic voltage-gated Ca2^+^ channel CaV2 in a dynamic manner. 2. CRMP2 SUMOylation is also thought to regulate membrane expression in the sodium channel NaV1.7. Syntaxin-1A SUMOylation is induced by NMDAR activation, resulting in decreased binding to SNAP-25 and serving as a key presynaptic modulator of vesicular endocytosis.

Sequencing of the whole exome of ASD patients identified a novel heterozygous protein truncation mutation in the Sentrin-specific peptidase 1 (SENP1) gene in individual autism patients. Pure mutations in the SENP1 gene are associated with severe neurological disorders, and SENP1 plays a key role in de-SUMOylation. Research has demonstrated that SENP1 haplotype deficient mice (Senp1+/−) exhibit social behavior deficits and increased stereotypic behavior. Interestingly, inhibitory and excitatory synaptic transmission was altered in layer II/III pyramidal neurons in the postsplenial granule-free (RSA) cortices of Senp1+/− mice. Mechanistically, SENP1 deficiency resulted in excessive SUMOylation and degradation of FMRP in the RSA [244]. Thus, SENP1 acts as a possible candidate gene for ASD dominance, serving as a circuit node involved in regulating mammalian social behavior (Figure 4).

### 4.5. Immunology and Neuroinflammation

Microbiota-gut-brain axis. The gut-brain axis comprises the whole gut microbiota, the enteric nervous system (ENS), the parasympathetic and sympathetic nervous systems, and the central nervous system. These structures interact functionally with the endocrine and immune systems through the involvement of cytokines, neuropeptides, and many other signaling molecules. It has been demonstrated that there is bidirectional communication of interactions between distinct linkages in the microbiota-gut-brain axis [245]. The microbiota has a direct impact on various aspects of nervous system function, including brain activity, blood-brain barrier permeability, neurogenesis synthesis, and non-transmitter excretion. The CNS has a substantial effect on the gastrointestinal tract and its microbiota. The nervous system regulates the secretion of intestinal peptides, gastric acid and mucus, intestinal permeability and mucosal immunity, influencing the habitat of intestinal microorganisms [246,247,248].

The gut, like the brain, contains an internal neuronal network called the ENS. The ENS oversees controlling the body’s main immunological organ. When intestinal immune cells are activated by infection, they provide responses to neurosensory circuits through efferent neuroimmune inflammatory reflex pathways. Over the last decade, research has revealed that ASD kids are more likely to develop gastrointestinal (GI) disorders. ASD Children are four times more likely than neurotypical children to develop GI [249]. Emerging studies have reported changes in the gut microbiota of ASD patients, with differences in microbiota abundance [250,251]. ASD patients are more likely to exhibit increased intestinal permeability, called “leaky gut” [252]. Germ-free mice colonized with intestinal bacteria from a pediatric ASD cohort are adequate to induce features associated with the autism phenotype. These mice’s brains also show alternative splicing of autism-associated genes. In addition, all currently known animal models of ASD exhibit some disruption in gut flora [71,253,254]. For example, increased intestinal permeability and altered intestinal flora have been observed in MIA mice; SHNAK3 deletion alters intestinal function and the gut microbiome [253,255]. The SHNAK3αβKO mice also exhibit alterations in gastrointestinal morphology and differences in fecal flora composition [255]. These mice also have higher hepatic LPS levels, higher IL-6 levels, and activated astrocytes [71]. Research on ASD animal models and ASD populations have shown that gut imbalance affects peripheral immune responses and leads to immune cell dysfunction.

Neuroimmune disorders. The immune system consists of a set of molecules and cells that interact closely with each other in tissues and organs. Adaptive immunity is crucial in neurodevelopmental disorders. Early evidence suggested that the immune system influences brain function in individuals with autism [256].

With the progressive understanding of neuroimmune, it has been found that neuroimmune crosstalk affects the connectivity of brain functions. Current studies suggest that cytokines secreted by neurons and glial cells further influence synaptic growth [257]. Multiple studies have found that microglia and astrocytes interact with synapses through surface ion channels, receptors, and transporter proteins in addition to inflammatory responses to regulate synaptic morphology and plasticity to maintain brain tremor sensation. Impairments in synaptic pruning and synaptic transmission have been observed in CX3C chemokine receptor 1 knockout mice. These defects may be due to increased IL-1β signaling secreted by microglia. Similarly, in fragile X syndrome mice, enhanced neuronal excitability was reported, presumably due to astrocytes influencing synaptic function and plasticity by regulating synaptic transmission via calcium signaling after glutamate uptake via the glutamate transport proteins GLAST and GLT1 [258]. On the other hand, MET and MHCI, as immune molecules and receptors, are closely associated with key functional defects in brain development. MET indirectly leads to changes in neural circuitry and function through negative regulation of immune responses and gastrointestinal homeostasis. MHCI molecules regulate synaptic pre- and postsynaptic areas related to glutamate homeostasis as well as axonal and synaptic development. In conclusion, dysregulation of the immunomodulatory signaling molecule crosstalk is critical in the pathogenesis of neuropsychiatric disorders like autism.

### 4.6. Internal Neural Loops in the Brain

Abnormal brain development in ASD patients is concentrated in the cerebral cortex, striatum, amygdala, and cerebellum. Recent studies have found that reduced excitatory synaptic transmission of pyramidal neurons in the prefrontal cortex causes autistic-like behaviors. The medial prefrontal cortex (mPFC) influences social behavior and neurally encodes spatial information [259]. Related reports also suggest that abnormal connections between the striatum and the cerebral cortex cause repetitive behaviors in autistic children [31,260]. In early assessments of autism, amygdala exhibits decreased volume and increased neuronal density, which is related to the modulation of critical functions in fear conditioning, anxiety, and social behavior [261]. The cerebellum is involved in the control of motor behavior, and most ASD patients have movement disorders as comorbidities. Furthermore, new research has revealed that cerebellar anomalies play a significant role in the onset of autism. A significant reduction of Purkinje cells was observed in the anatomy of autistic patients. Knockdown of the critical molecular signals such as TSC1, TSC2, and Bmal1 in the PC of the cerebellum causes altered core behaviors in ASD [262,263]. Kelly et al. identified a novel inhibitory circuit between the cerebellar nucleus, ventral thalamus, and MPFC involving the cerebellar cortical region right CRUS 1 (RCRUS1). Disruption of this circuit led to social deficiencies and repeated behaviors. In the large and complex neural network affecting social behavior, the PFC and its large number of reciprocal circuit connections constitute a top-down system that controls the norms of social behavior [264]. The continuous refinement of this system offers the potential for finding neuromodulatory targets for ASD therapy.

## 5. Targeting Molecules in Neural Circuitry May Be the Prospect of Autism Spectrum Disorder Treatment

**Figure 5:**
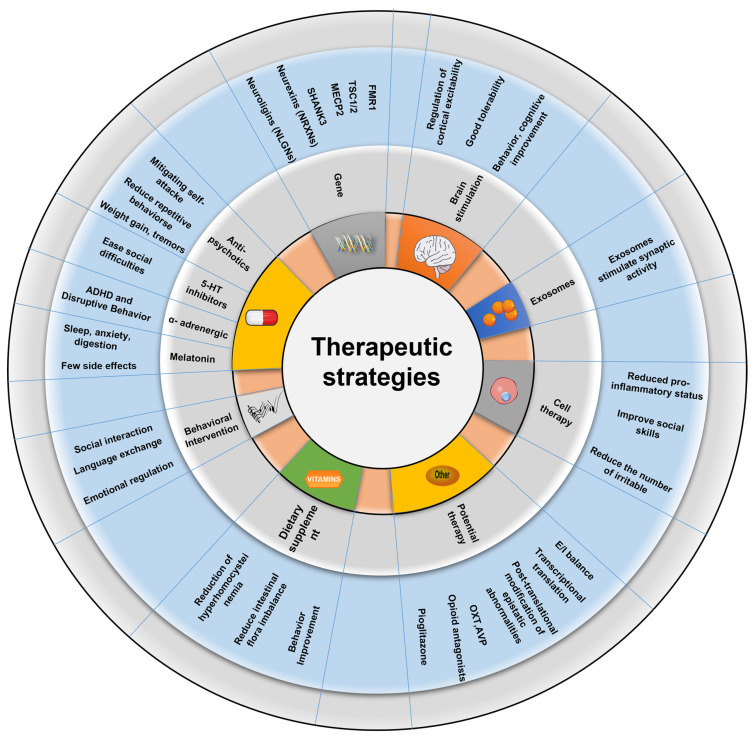
Potential therapy strategies for autism spectrum diseases. The small gray circles represent components of current ASD treatment strategies. Pharmacological treatments, nonpharmacological treatments, cellular and exosomal therapies, gene therapy, and potential emerging therapeutics are all covered. The large blue circles represent the benefits and drawbacks of various ASD therapy options for specific symptoms. Specific developments in emerging treatment methods are also discussed.

Autism treatments are now classified as nonpharmacological or pharmacological. Although nonpharmacological therapy methods show some promising results, their therapeutic benefits are limited. Current medications address some of the common symptoms of ASD, however they are ineffective for the core symptoms, which include impaired communication and social interaction, as well as the presence of restricted and repetitive behaviors. Based on the overlay of multiple complex etiological mechanisms in autism, combining novel drug development therapies with behavioral interventions may have a significant impact on individuals with autism in the future. Currently, the lack of appropriate regional and molecular targets in the study of new drugs for autism makes the discovery of new drugs for the treatment of autism the most innovative task (Figure 5).

### 5.1. Nonpharmacological Therapies

The prevalence of ASD has increased year by year and available clinical medications have become scarce, nonpharmacological treatments including educational interventions, behavioral modification primary measures supplemented by music therapy, and brain stimulation have been used to improve social skills and enhance the life ability of ASD patients.

Behavioral psychological treatments. In young children with autism, behavioral psychological treatments are preferred. Music treatment, cognitive and social behavioral therapy, which are currently widely used, have demonstrated some improvement in social interaction in verbal language communication in people with autism [265]. Music treatment in the early neurodevelopmental stage can achieve the integration of cortical and subcortical regions by altering cortical structures and functional connections. Cognitive behavioral therapy (CBT) has advantages for treating core symptoms and comorbidities, such as anxiety and depression in ASD patients because of its highly organized and predictable character. Social behavioral therapy (SBT) focuses on developing functional independence in individuals with ASD via emotional regulation, social skills, and communication [266].

Non-invasive brain stimulation. Non-invasive brain stimulation is a novel therapeutic approach. It modulates local cortical excitability, which influences cell excitability and synaptic plasticity. Transcranial magnetic stimulation (TMS) and transcranial direct current stimulation (tDCS) are the two major components of the method. tDCS is primarily performed in the brain using a continuous current delivered by scalp electrodes. In TMS, a fluctuating extracranial magnetic field induces intracranial currents in the brain. Children and adults accept both strategies well [267]. Recent studies have shown improvements in social behavior and cognition in ASD patients treated with TMS or tDCS [268,269].

Dietary supplement therapy. Research studies have reported that certain vitamins (e.g., vitamin B6, B12 and D; folic acid) [270,271], omega-3 polyunsaturated fatty acids (PUFAs) [272], probiotics [273], and certain chemicals from plants (e.g., lignans and radicicicol) have an effect on reducing hyperhomocysteinemia and gastrointestinal problems, as well as improve ASD symptoms. Several studies have also reported positive results linked with general dietary treatments, such as gluten-free and casein-free diets, which are thought to improve GI function, reduce gut flora dysbiosis, and improve some ASD behavioral symptoms [274]. However, the efficacy and safety of such dietary therapies need to be confirmed by further investigations.

In conclusion, nonpharmacological therapies can partially alleviate symptoms of autism. Despite the absence of sufficient data, the therapeutic effects of behavioral and psychological treatments, brain stimulation, and dietary therapies for individuals with autism appear to have a theoretical basis in neurobiochemistry and signal transduction.

### 5.2. Pharmacological Therapies

To date, no drugs are available to treat the core defects in patients with ASD, and currently pharmacological therapies are only used to address adverse adaptive behaviors and comorbidities such as sleep and anxiety that cannot be controlled by behavioral therapy [275].

Aripiprazole and risperidone are currently approved for the treatment of ASD and they can alleviate people with ASD with their self-aggressive, irritable behavior [276]. Aripiprazole was observed to reduce self-aggression, irritability, and repetitive stereotypic behaviors in two short-term randomized controlled studies. However, common side effects were sedation, tremor, salivation, and weight gain. Risperidone was shown to be effective and well tolerated in the short- and long-term treatment and discontinuation phases, with one in three children/adolescents with ASD showing behavioral improvement. Risperidone treatment can lead to weight gain [277]. Because of the Hyperhydroxytryptophanemia frequently observed in children with ASD, selective serotonin reuptake inhibitors (SSRIs) have been used to treat anxiety and depression and alleviate social defects by blocking 5-HT reuptake and increasing the amount of 5-HT in synaptic cleft in children with ASD [278,279]. Guanfacine, is used to treat ADHD and disruptive behavior [280]. In addition, melatonin can reduce sleep-related problems in children with ASD and can also improve symptoms such as pain, depression, anxiety, depression, and gastrointestinal dysfunction with few adverse effects [281,282]. Thus, melatonin or its derivatives may become the most promising drugs therapies to improve behavioral disorders in autism patient.

### 5.3. Cell Therapies

Cellular therapies, notably bone marrow hematopoietic and mesenchymal stem cell transplantation, have seen increasing use in the treatment of neurological problems. Hematopoietic stem-cell therapy can prolong the life of children with ASD by preventing neurodegeneration [283]. Stem cells reduce the pro-inflammatory state by synthesizing and releasing chemokines, cytokines, and growth factors, which influence the expression of inflammatory markers in the blood of children with ASD [284]. Moreover, stem cells can induce the recruitment and differentiation of native stem cells. Twelve months after single intravenous infusions of autologous cord blood were administered to each of 25 children with ASD, their electroencephalography (EEG) spectra showed significant changes including increases in alpha and beta power and a decrease in EEG theta power, accompanied by an improvement in the patients’ social communication skills and ASD symptoms [285,286]. Fetal stem-cell transplantation by intravenous and subcutaneous injections in ASD patients (3–15 years old) improved social skills in the treatment group with no adverse effects. In 37 children with ASD, the transplant group showed a reduction in stereotypic and agitated behavior, and higher scores on the autism rating scale. Several interventional clinical trials evaluating the impact of stem-cell therapy on children with ASD are underway.

### 5.4. Neurotransmitter Manipulations

E/I imbalance is a key reason for the pathogenesis of ASD patients, and modulators targeting GABA and glutamate receptors have been developed in order to restore the E/I balance [287]. In mice models of autism induced by mGluR5 overactivity, mGluR5 antagonists have been applied to treat social deficits, learning memory deficits, repetitive stereotypic behavior, and dendritic spine abnormalities [288]. Unfortunately, large-scale patient studies on mGluR5 inhibitors for fragile X syndrome have reported negative results [289]. NMDAR agonists mainly correct the aberrant transmission of excitatory synaptic signals, and were observed to rescue some of the impaired social competence in Shank2 transgenic mice [290]. Memantine, one of the NMDARs, can improve stereotypic behavior and social deficits [291]. Other NMDA-modulating drugs such as d-cycloserine, ketamine, and riluzole have shown negative results [292,293]. A larger sample size of studies is required to confirm the therapeutic effect of these drugs. GABAR agonists correct abnormal GABA-mediated synaptic transmission, which helps to alleviate some of the social deficits shown in animals with fragile X syndrome [294]. Abaclofen inhibits glutamate release to affect the phenotype of protein synthesis and reverse synaptic abnormalities [295]. Two-phase clinical trials have already suggested that abaclofen can improve ASD symptoms [296]. Bumetanide, an NKKCC1 chloride input inhibitor, enhances individual ASD behavioral features by increasing GABAergic inhibition caused by decreasing chlorine ion levels. Follow-up data demonstrated that bumetanide improves symptoms in ASD patients [297].

### 5.5. Targeted Translation and Epigenetic Regulation

Studies in transcriptional and translational levels provide a scientific basis for discovering potential mechanistic drug targets. TSC patients have been treated with mTOR inhibitors for behavioral and molecular abnormalities [298]. Preliminary studies have shown that IGF-1 increases synaptic protein levels and changes dendritic spine density and excitatory synaptic transmission in cortical area via activating the MAPK and mTOR pathways. IGF-1 has been found in clinical studies to be beneficial in the treatment of ASD patient. PPAR γ agonists activate Dickkopf-1 activity, inhibit the Wnt/-Catenin pathway, and improve behavioral deficits. In terms of epigenetic regulation, numerous autism risk genes are engaged in abnormal epigenetic post-translational modifications. Therapeutic strategies for epigenetic enzymes focus on therapies targeting enzymes at different stages of the post-translational modification process, resulting in modest improvement in autistic mouse behavior. Studies using SHANK3 mutant mouse models have found that histone methyltransferases and histone acetylase inhibitors, alone or in combination, significantly improve synaptic dysfunction and social interaction [299,300,301].

### 5.6. Other Biological Targets

Animal studies back up the neuropeptide theory of autism. Oxytocin (OXT), as a neuropeptide, plays an important role in many physiological processes. It affects social behavior by regulating neuronal plasticity [302]. OXT plasma levels have been reported to be altered in autistic patient, and they are frequently related to dysfunctional connectivity [303]. Animal studies have indicated that OXT treatment can rescue autism-associated social deficits [304]. Evidence supported that oxytocin administration reduces some dysfunctional behaviors associated with autism, including social performance, anxiety behaviors etc. The arginine vasopressin (AVP) is a member of the same superfamily as OXT. Blinded clinical trials have shown that intranasal AVP in children can improve defects in social competency, and it has been used as a target for ASD drug treatment [305,306]. Several studies have found that opioid antagonists can reduce self-aggression, ADHD, and agitated behavior in people with autism. However, the evidence is lacking that they can improve core autism symptoms in most participants. Pioglitazone is a thiazolidinedione that acts on the peroxisome proliferator-activated receptor (PPAR-Y, a nuclear hormone receptor). Pioglitazone has also been shown to reduce NMDA-mediated Ca2+ currents and transients. Two clinical trials have demonstrated the potential of pioglitazone to improve behavioral symptoms of ASD [307].

## 6. Conclusions and Future Perspectives

Although the incidence of ASD is increasing, there is no physical or pharmacological treatment for ASD, and the etiology is highly complicated, involving genetic mutations, maternal immune activation, and environmental triggers. Ultimately, molecular signaling pathways, neuronal synapses, epigenetic post-translational modifications, immune activation, and connectivity abnormalities in brain function are induced. Thus, different animal models both increase our understanding of genetic and environmental factors underlying the onset of ASD and offer new potential ideas for treatment. Here, we explore recent advances in pathological studies of different animal models to cause ASD-like behavior in experimental animals by ameliorating or reversing neuronal damage caused by genetic, inflammatory, and prenatal drug exposure. Multidimensional analysis of behavioral characteristics and neuropathological alterations in autistic mice and autistic populations attempts to connect animal studies to ASD patients. We can use the data from animal studies on ASD symptoms to explore abnormalities at the biomolecular level in models of autism to find relationships with behavioral regulation and further discover key molecular targets for signaling pathways. So far, some of the available treatments, including drugs and non-drugs appear to relieve some of common symptoms but are ineffective in treating the core symptoms of ASD. In the search for the ultimate intervention to improve ASD treatment, the development of effective therapies still require a further in-depth study of the disease’s core defects and normalization of its pathophysiology.

Autism is a behavior-based disorder. While diagnostic criteria strive to maximize clinical consensus, multilevel analyses do not always reflect its extensive inter- and intra-individual heterogeneity. Given the heterogeneity of ASD, no single model can generalize all symptoms. By manipulating non-human primate genome alterations to mimic the pathological process of ASD, in-depth mechanistic studies are extremely important for clinical translation. Hundreds of genes have been defined in genetic studies as risk factors for development of autism. While the functions of many genes remain obscure, the proteins encoded by the genes found so far participate in three common pathways: translational regulation, transcriptional/epigenetic regulation, and synaptic development/plasticity regulation. New developments in gene therapy, such as gene replacement, CRISPR-Cas9 gene editing, oligonucleotide translation, etc., have to some extent fueled the development of gene therapy as a new strategy for personalized treatment [308]. Attention to potential links between biochemical molecular systems, neural circuits, and environmental variables is necessary to optimize therapeutic approaches for autism. Multiple clinical disorders frequently coexist in autism, however, current research has paid inadequate attention to relevant comorbidities. The search for potential biomarkers should be combined with assessment of the presence or absence of specific comorbidities when designing experiments related to specific clinical syndromes. These should be combined with large-scale population-based cohort studies to identify the dynamic spatial and temporal links between behavior, development, and comorbidity types. In conclusion, the study of ASD remains challenging, and mechanistic treatments of these disorders will only succeed when the heterogeneity of neurodevelopmental disorders is incorporated into precision medicine through multidisciplinary integration.

## Figures and Tables

**Table 1:** Mouse models of ASD.

Category	Gene Symbol	Molecular Function/Copy Number	Molecular, Cellular and Circuit Phenotypes	Refs
Genetic animal models	NRXNs	Synaptic adhesion molecule	↓Glutamatergic trans. and synaptic density	[7,8,9,10]
	NLGNs	Synaptic adhesion molecule	Context-dependent impaired glutamatergic and GABAergic trans; ↓Brain volume, cerebellar deficit; ↓GABAergic trans. in D1-MSN in NAc; ↓Brain volume	[11,12,13,14]
	SHANK3	Synaptic scaffolding molecule	Striatal dysfunction; ↓Activity-dependent AMPAR distribution and LTP; ↓Glutamatergic trans. by presynaptic mechanism, ↓LTP; ↓NMDAR function, Rac1, PAK, cofilin signaling defects, F-actin dysregulation in PFC	[15,16,17,18,19]
	TSC1/2	Translational regulator	Cerebellar deficits; Brain enlargement, hyperactive mTOR signaling, autophagy deficiency	[20,21,22]
	FMR1	Translational regulator	↑mGluR function, immature protrusion; PI3K signaling, ↑spine density, impaired AMPAR-mediated synaptic plasticity; Hypersensitivity to ERK1/2 pathway activation, ↑protein synthesis; ↑Fetal or early postnatal GABA and Cl–, abnormal EEG	[23,24,25]
	MECP2	Translational regulator	↓Dopamine transporter (DAT) and tyrosine hydroxylase (TH) in the striatum; Altered cortical and cerebellar volumes; Cortical LTP deficit; ↓cortical BDNF levels; Impaired PI3K/AKT/mTOR pathway; ↓CB1 and CB2 receptor levels; Hippocampal circuit dysfunction	[26,27,28,29]
	CHD8	Translational regulator	CHD8 regulates different sets of genes associated with ASD by direct and indirect mechanisms	[30,31,32]
	SCN1A	Na^+^ channel	↓GABAergic interneuron firing	[33]
	SYNGAP1	Alternative splicing	Major constituent of the PSD essential for postsynaptic signaling; SYNGAP1 regulates the postmitotic maturation of human neurons made from hiPSCs, which influences how activity develops within nascent neural networks	[34,35]
	ADNP	Translational regulator	Potential transcription factor; mediate some of the neuroprotective peptide VIP-associated effects involving normal growth	[36]
	ANK2	Protein transport	endocytosis and intracellular protein transport; continuous directional cell migration	[37]
	CUL3	Transport	Mediate ubiquitination of target proteins; releases the GATOR1 complex-mediated inhibition of the TORC1 pathway	[38]
	PTEN	Apoptosis	Neuron positioning, dendritic development and synapse formation	[39]
	TBR1	Transcription	Neuronal migration, laminar and areal identity, and axonal projection; blocks the formation of the corticospinal (CS) tract from layer 6 projection neurons	[40]
	SCN2A	Ion transport	Mediates the voltage-dependent sodium ion permeability of excitable membranes	[41]
	TRIP12	DNA repair	Ubiquitin fusion degradation (UFD) pathway and	[42]
			regulation of DNA repair	
	RELN	Cell adhesion	Regulates microtubule function in neurons and neuronal migration; affects migration of sympathetic preganglionic neurons in the spinal cord	[43]
	UBE3A	Proteolysis	Acts as a regulator of synaptic development by mediating ubiquitination and degradation of ARC; Synergizes with WBP2 in enhancing PGR activity	[37]
	Cntnap2	Cell adhesion	Plays a role in the formation of functionally distinct domains critical for saltatory conduction of nerve impulses in myelinated nerve fibers	[44]
	Grin2b	Ion transport	Irreversible neuronal death; neural pattern formation; long-term depression (LTD) of hippocampus membrane currents and in synaptic plasticity	[45,46]
Copy number variation	15q11-q13	Deletion	↑excitatory synaptic event frequency amplitude, density of dendritic protrusions,↓inhibitory synaptic transmission; Impaired activity-dependent synaptic plasticity and homoeostatic synaptic scaling	[47,48]
	15q13.3	Microdeletion	↑endoplasmic reticulum stress; Dysregulated neuronal gene expression; ↑cholinergic activity; ↑homomeric CHRNA7 channel activity	[49,50]
	16p11.2	Deletion	↑soma size and dendrite length in 16pdel neurons; ↓neuronal size and dendrite length in 16pdup neurons; ↓synaptic density	[51,52]
	22q11.2	Duplication	↓spontaneous neuronal activity and calcium signalling; ↓expression of miR-1290	[53]
Idiopathic animal models	BTBR-T+ tfl/J		↓GABAergic inhibitory transmission; ↓5HT2A receptor density and activity; ↑glutamatergic transmission in cortico-striatal circuitry; Impaired dopamine D2 receptor function; ↓BDNF expression in hippocampus and cortex; Absence of corpus callosum, lack of hippocampal commissure; ↓cortical thickness; ↓cerebral white and gray matter; Impaired cortico-thalamic function; Altered volumes of cerebellum, brainstem, striatum, and hippocampus	[54,55,56,57,58,59]
	BALB/cByJ		Interleukin change; neuroinflammation	[60]
Environmental models	VPA		↑glutamatergic excitatory signaling; Hyperexcitable local connectivity;↓parvalbumin-positive inhibitory interneurons;↑brain serotonin levels; Apical dendritic arborization complexity;↓PTEN expression and↑p-AKT protein levels in hippocampus and cortex	[61,62,63,64]
	Maternal immune activation		Changes of various interleukins in brain	[65]

## Data Availability

Not applicable.
